# Epidermal growth factor receptor function in the human urothelium

**DOI:** 10.1007/s11255-018-1831-z

**Published:** 2018-03-05

**Authors:** C. Wasén, M. Ekstrand, M. Levin, D. Giglio

**Affiliations:** 1 Department of Pharmacology, The Sahlgrenska Academy, University of Gothenburg, Box 431, 405 30 Göteborg, Sweden; 2 The Wallenberg Laboratory, The Sahlgrenska Academy, University of Gothenburg, Göteborg, Sweden; 3 Department of Oncology, The Sahlgrenska Academy, University of Gothenburg, Göteborg, Sweden

**Keywords:** Urothelium, Proliferation, Epidermal growth factor receptor, Occludin, Three-dimensional cell culture

## Abstract

**Purpose:**

Epidermal growth factor receptor (EGFr)-targeted therapy may be used in subgroups of patients with urinary bladder cancer. Here we assessed the role of EGFr in urothelial proliferation and migration in a two- and three-dimensional cell culture system.

**Methods:**

UROtsa cells derived from normal urothelium and malignant T24 cells were cultured in a Type I collagen gel. Proliferation and migration of urothelial cells, in the absence and presence of the EGFr inhibitor cetuximab, were assessed with a proliferation test (ATCC) and with the Axioplan 2 imaging microscope with a motorized stage (Carl Zeiss), respectively. The expressions of cytokeratin (CK) 17, CK20, EGFr, pEGFr, laminin, occludin and zonula occludens 1 (ZO-1) were assessed with immunohistochemistry and/or western blot.

**Results:**

UROtsa spheroids were formed after 7 days in culture, while T24 cells did not form spheroids. UROtsa expressed CK20 but not laminin or CK17 and consequently resembled umbrella cells. In UROtsa and T24, cetuximab inhibited urothelial proliferation, induced cleavage of EGFr and/or pEGFR but did not affect urothelial migration. The tight junction protein occludin was cleaved, and the formation of cellular spheroids was inhibited in UROtsa by the presence of cetuximab.

**Conclusions:**

EGFr modulates urothelial proliferation and the formation of the three-dimensional structure of the urothelium possibly by interfering with occludin. The present data also show a cell culture technique enabling phenotypically normal urothelial cells to form epithelial structures in contrast to malignant urothelial cells.

## Introduction

The epithelial lining of the urinary bladder, the urothelium, is the origin of the predominant part of all tumors in the urinary tract. At present, there is a lack of knowledge about how urothelial proliferation and migration are regulated and what leads to transformation and tumor development. Normally, urothelial cells have a low proliferation rate, but in pathological conditions, such as in bladder inflammation, in malignancy and after different stimuli, urothelial proliferation may increase dramatically. Epidermal growth factor receptor (EGFr) plays a pivotal role for cellular proliferation and migration in different tissues. EGFr is expressed in the urothelium and seems here also to play a role for urothelial proliferation and migration [1–3]. Studies show that radiation toward the urinary bladder and cyclophosphamide treatment in rodents may trigger urothelial proliferation concomitant with an enhanced urothelial expression of EGFr [1, 4]. EGFr may also be involved in the pathogenesis of urinary bladder cancer, i.e., overexpression of EGFr in urinary bladder tumors has been shown to be associated with increased mortality [5]. A large portion of bladder tumors also over-express EGFr [6]. Medical trials have, however, not shown effectiveness when testing EGFr blockade in the therapy of urinary bladder cancer [7, 8]. Blockade of the EGFr pathway may yet be putatively effective in bladder cancer therapy in subgroups of patients with basal muscle-invasive bladder cancer with an activated EGFr signaling pathway [9, 10]. This highlights the importance to screen for mutations to optimize cancer treatment.

In the present study, we assessed EGFr signaling in the normal urothelium and in bladder cancer by letting normal and malignant cells grow in conventional cell cultures and in three-dimensional cell cultures. In contrast to conventional cell cultures where cells normally form only a one-cell layer, three-dimensional cell cultures form multilayered structures and sometimes also form entities such as liver lobules [11]. Three-dimensional growth of cells allows analysis of many functions of the cell including proliferation, migration and differentiation with better resemblance to the in vivo situation than conventional cultures. The focus of this study was to assess the role of EGFr in proliferation and migration of urothelial cells. Two urothelial cell lines with contra-polarized characteristics were presently compared, i.e., the UROtsa and the T24 cell line. The UROtsa cell line is an immortalized normal human bladder urothelial cell line derived from a 12-year-old girl and is not neoplastic, i.e., it does not induce tumors in naked mice [12]. The urothelial human bladder cancer cell line T24 is derived from a highly malignant grade III human urothelial cell carcinoma of a woman [13].

## Methods and materials

### Cell cultivation

UROtsa cells and T24 cells were grown according to instructions of the manufacturer, i.e., UROtsa in Dulbecco’s modified Eagle medium (DMEM; Sigma-Aldrich, St. Louis, MO, USA) and T24 in McCoy’s 5a medium (LGC Promochem, Borås, Sweden) with the addition of 10% fetal bovine serum (Sigma-Aldrich) and 1% penicillin/streptomycin (PEST; PAA Laboratories, Pasching, Austria). UROtsa and T24 cells were grown at a temperature of 37 °C and at an atmosphere of 5% CO_2_. Medium renewal occurred every 2–3 days. UROtsa and T24 cells used in the different experiments were all used at similar passages, i.e., passages 10–25.

### Immunocytochemistry of 2D cultures

For immunocytochemical studies, UROTSA and T24 cells were grown on thin cover slides in cell culture plates at a concentration of 100,000 cells/well and 50,000 cells/wells, respectively, and fixated with 4% formaldehyde solution (confluency of 50–60%) 3 days later. After washes in phosphate-buffered saline (PBS), cells were blocked with PBS containing 0.7% fish skin gelatin (PFS; gelatin from cold-water fish skin, Sigma-Aldrich) for 40 min. After washes in PBS, cells were incubated for 3 h with the primary antibody (see list below) diluted in PFS. Cells were washed with PFS and PBS and then incubated with the secondary antibody (1:250 in PFS; see list below) overnight at 4 °C. On the next day, cells were washed with PBS, and then, ProLong^®^ Gold Antifade Reagent with DAPI (Life technologies Ltd, Paisley, UK) was put onto the cells on the cover glasses. The cover glass was then turned and put on a glass slide. As a negative control, the protocol was also performed as above without cells being exposed to the primary antibody, which resulted in no immunostaining of the cells.

### Western blot

Urothelial cells were cultured for 3 days and then lysed in lysis buffer, i.e., HBSS buffer containing 0.1% phosphatase inhibitor cocktail 2 (Sigma-Aldrich), 0.5% protease inhibitor cocktail (Sigma-Aldrich), triton X-100 and deoxycholate (Sigma-Aldrich). Lysed cells were frozen to − 70 °C and thawed twice, followed by centrifugation at 10,000 rpm at − 4 °C, and the supernatants were then maintained. The Pierce BCA Protein Assay Kit (Thermo Scientific, Rockford, IL, USA) was used to determine the protein concentrations. Protein samples were then mixed with NuPAGE LDS Sample Buffer (Life Technologies) and NuPAGE Reducing Agent (Life Technologies) and boiled for 5 min followed by loading 5 μg of protein per sample per well on a NuPAGE 4–12% Bis–Tris gel (Life Technologies). Proteins were separated by electrophoresis at 200 V under reducing conditions in MOPS buffer (Life Technologies) followed by transfer of the proteins onto nitrocellulose membranes (Life Technologies) for 60 min at 30 V. The membranes were thereafter washed in Tris-buffered saline containing 0.3% Tween 20 (TBS-T; Sigma-Aldrich) and then blocked for 60 min with TBS-T containing 5% nonfat milk. The membranes were incubated overnight with the primary antibody (see list below) diluted in TBS-T containing 3% goat serum (Sigma-Aldrich). The following morning, after washes in TBS-T, the membrane was incubated with the secondary antibody (see list below) diluted in TBS-T containing 5% nonfat milk for 1 h. After washes in TBS-T, the binding of the antibody was detected with Amersham ECL Plus™ Western Blotting System (GE Healthcare, Little Chalfont, UK) visualized by using the Fujifilm Image Reader LAS-1000 Pro v.2.6 (Stockholm, Sweden). Quantification of pixel intensity was made with the Fujifilm Multi Gauge v3.0 software (Stockholm, Sweden). Membranes were stripped in Restore Western Blot Stripping Buffer (Thermo Scientific) and incubated with other primary antibodies followed by same procedure as described above. Membranes were also incubated with anti-EGFr in the presence of EGFr peptide.

### Three-dimensional cultures

Urothelial cells (UROtsa and T24) were diluted in their respective medium at a concentration of 50 million cells/ml. Consequently, the cells were mixed with a gel containing Type I collagen (84%; Advanced Bio Matrix), l-glutamine (7.8%; Sigma-Aldrich), sodium bicarbonate 7.5% (2.1%; MP Biomedicals LLC, Solon, Ohio, USA) and 10 × Minimum Essential Medium (10 × MEM; Invitrogen) 6.5%; to a final cell concentration in the gel of 500,000 cells/ml. Cell culture was performed in 12-filter culture dishes (Transwell Permeable Supports, Costar, USA; product # 3460). Before adding the cells to the culture dishes, the filters of the dishes were coated with cell-free Type I collagen gel suspension and were allowed to solidify at 37 °C; 250 μl of the cell-gel suspension was then added to the coated filters, and the culture dish was again placed in an incubator at 37 °C for the gel to solidify for 30 min. The cell-gel suspension was grown in DMEM (UROtsa) or McCoy’s 5a medium (T24) in an incubator at 37 °C (5% CO_2_). Medium renewal occurred every 2–3 days.

### Immunocytochemistry of 3D cultures

The urothelial cells were either fixated after 7 or 14 days after culture. On the day of fixation, cells were first treated with collagenase (100 U/ml) to make the cells readily available for staining with antibodies. After washes in PBS, cells were fixated with 4% formaldehyde solution for 30 min. Cells were then blocked with PBS containing 0.7% fish skin gelatin and 0.03% saponin (Sigma-Aldrich) for 40 min. Antibodies and phalloidin were diluted in PFS. Gels were consequently incubated with various primary antibodies (see list below) and co-stained with rhodamine phalloidin (1:50; Life technologies Ltd) in order to stain F-actin. Gels were incubated with the primary antibody (see list below) in 6-well plates (Sarstedt; Newton, USA) for 3 h at room temperature. Gels were then washed in PFS followed by incubation with the secondary antibody (see list below) for 24 h at 4 °C. As a negative control, the protocol was performed as above without cells being exposed to the primary antibody, which resulted in no immunostaining of the cells. UROtsa cells were also grown three-dimensionally in the absence and presence of cetuximab (1.5 μM) for 14 days. Gels were washed and mounted on glass slides with ProLong^®^ Gold Antifade Reagent. The number of created cysts in the absence and presence of cetuximab was counted blindly in four regions of each 3D culture, and the mean number of spheroids in each culture was then calculated.

### List of antibodies and antipeptides (IHC: immunohistochemistry; WB: western blot; concentration)

Alexa Fluor^®^ 488 goat anti-rabbit IgG (H + L) * 2 mg/ml (IHC 1:250; Life technologies Ltd; A11008), Alexa Fluor^®^ 488 goat anti-rat (IHC 1:250; Life technologies Ltd; A11006), EGFR peptide (Abcam, ab204282), goat anti-mouse IgG (H + L) − HRP (WB 1:4000; Life Technologies; 62-6520), goat anti-rabbit IgG H&L (WB 1:5000; Abcam; ab6721), mouse anti-β-actin (WB 1:2000, SCBT, sc-4778), mouse anti-cytokeratin 17 (IHC 1:100; Sigma-Aldrich (SA)), rabbit anti-cytokeratin 20 (IHC 1:100; SA), rabbit anti-EGFR (WB: 1:1000; Abcam, ab52894), rabbit anti-EGFr (IHC: 1:100; Santa Cruz Biotechnology (SCBT), Santa Cruz, CA, USA; sc-03), rabbit anti-occludin (WB 1:500; ThermoFisher Scientific; 71-1500), rabbit anti-pEGFr (WB 1:1000; phosphorylated Y1068; Abcam; Ab5644), rabbit zonula occludens 1 (WB 1:500; ThermoFisher Scientific; 40-2200), rat anti-laminin (IHC 1:100; Abcam, Cambridge, MA, USA) and Texas Red^®^ goat anti-mouse IgG H + L (IHC 1:250; Life technologies Ltd).

### Proliferation test

UROtsa and T24 cells were trypsinized and thereafter resuspended in medium at a concentration of 400,000 cells/ml (24 h in culture) or 50,000 cells/ml (72 h in culture); 100 μl of aliquots of cell suspension was then added in each well of a 96-well plate. Cells seeded were allowed to adhere for 1 h in the incubator at 37 °C prior to adding cetuximab. Cells were then incubated with cetuximab (1.5 μM; anti-EGFR IgG1 mAb, Merck Serono, Darmstadt, Germany) or PBS (control) for 24 or 72 h, respectively. The total volume of antagonists added to each well was 10 μl. The MTT Cell Proliferation Test was performed according to the instructions of the manufacturer (ATCC, Manassas, VA, USA). Thus, after 24–72 h of cell culture, urothelial cells were incubated with yellow MTT (3-(4,5-dimethylthiazol-2-yl)-2,5-diphenyltetrazolium bromide) for 3.5 h. Yellow MTT is metabolized to purple formazan depending on the activeness of reductase enzymes in the mitochondria. Thereafter, cells were exposed to a detergent overnight in order to lysate the cells. The intensity in color is proportional to the number of viable cells in the sample and was measured with a spectrophotometer (measured at 570 nm).

### Time-lapse filming and analyses of migration

For migration studies, urothelial cells were cultured on TPP tissue plate 60 (TPP Techno Plastic Products AG, Trasadingen, Switzerland) inside PC4L-0.5-CoverWell Perfusion Chambers (Grace Bio-Labs Inc, Bend, Oregon, USA) at a concentration of 100,000 cells/ml in culture medium containing 20 mM HEPES (1 M, H0087, Sigma-Aldrich), and sealed with two 22 × 22 mm coverslips and melted VALAP (1:1:1 weight of vaseline lanolin, paraffin wax). Cells were then incubated with cetuximab (1.5 μM) or PBS (control). The plate was put in a thermostatic cell incubation chamber for 1 h to allow cell attachment, after which the plate was inverted and an Axioplan 2 imaging microscope with a motorized stage (Carl Zeiss Microscopy GmbH) was used to assess migration. Four representative spots per well were selected, and the cells were photographed every 5 min for 48 h using a macro in KS 400 (version 3.0, Carl Zeiss Vision GmbH). The resulting time-lapse images were made into videos using Images to Video (version 3.0.0, Jaromir Sivic).

Films were analyzed using the image analysis software ImageJ (Rasband, W.S., ImageJ, US National Institutes of Health, Bethesda, Maryland, USA, http://imagej.nih.gov/ij/, 1997–2014) and the cell tracking plugin MTrackJ (created by Erik Meijering; www.imagescience.org/meijering/software/mtrackj). Five cells per film were selected and tracked frame by frame in order to measure the mean velocity of these cells. The paths of the tracked cells were unknown at the time of selection, and 2–3 cells that were alone and 2–3 cells that were part of a small cell island were chosen and tracked per film. The analysis was blinded. The mean velocity of the five cells was calculated, representing the mean velocity of each well, and these values were used to calculate the mean velocity of each treatment group. An area of each film covering 150–200 cells was monitored, and the number of cells within this area was counted at 120, 520 and at 920 min after seeding. Moreover, the percentage of cells forming mitotic rounding at these time points was calculated [14].

### Data analysis and statistical calculations

All data values are expressed as mean ± SEM. Statistical significance was determined by the Mann–Whitney test. *p* values of 0.05 or less were regarded as statistically significant. Graphs were generated, and parameters computed using the GraphPad Prism program (GraphPad Software, Inc., San Diego, CA, USA).

## Results

**Fig. 1:**
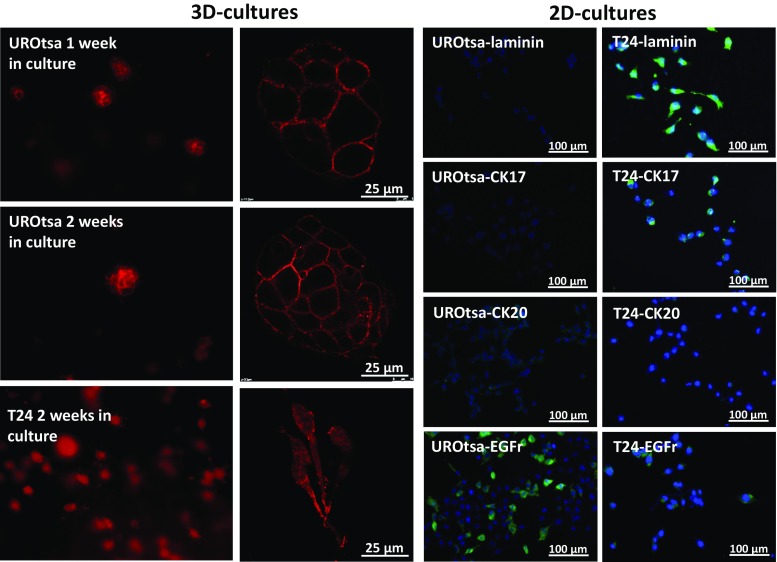
First (fluorescence microscopy) and second (confocal microscopy) columns represent UROtsa grown for 1 and 2 weeks and T24 grown for 2 weeks. Cells were stained with phalloidin. Representative microphotographs of the expressions of laminin, CK17, CK20 and EGFr (green) with DAPI-stained (blue) nuclei in UROtsa (third column) and T24 (fourth column)

Cultivating UROtsa cells in Type I collagen gave rise to three-dimensional multi-cellular cyst formations of around 50 μM after 7 days and around 75 μM after 14 days (Fig. 1). Beyond this time point, the spheroids did not grow in size, but cells were still alive after 30 days in culture. In order to determine the epithelial cell character of the two chosen urothelial cell lines, immunofluorescence was performed on different markers for cells within the urothelium. Two-dimensional cell cultures of UROtsa cells and T24 cells showed different patterns in the expression of markers for umbrella cells *vs.* basal and intermediate cells, i.e., UROtsa cells expressed CK20, low levels of laminin but did not express CK17 (Fig. 1). T24 cells expressed instead CK17 and laminin but low levels of CK20 (Fig. 1). Two-dimensional cell cultures of UROTSA and T24 cells showed that the expression of EGFr predominantly occurred in dividing cells (Fig. 1).

### Effects of cetuximab on proliferation and migration of urothelial cells

**Fig. 2:**
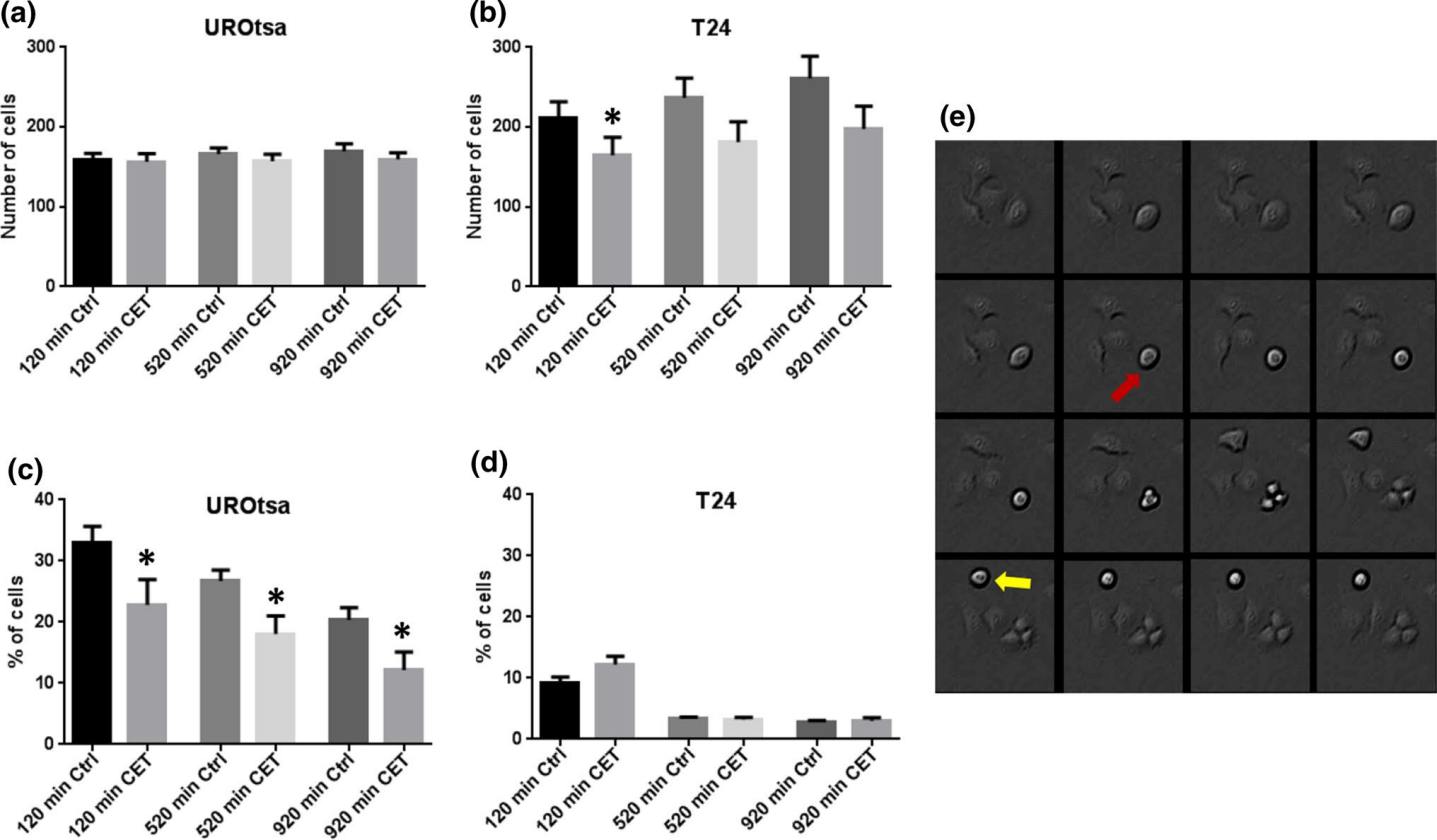
Total number of cells per vision field in **a** UROtsa and **b** T24 and percentages of cells with round shapes out of the total number of cells per vision field in **c** UROtsa and **d** T24 in the absence and presence of cetuximab (1.5 μM) after 120, 520 and 920 min after cell culture and **e** representative time-lapse series (320 s between frames) of T24 cells undergoing tripolar mitosis (red arrow) and regular dipolar mitosis (yellow arrow). *p < 0.05. Vertical bars represent the SEM

**Fig. 3:**
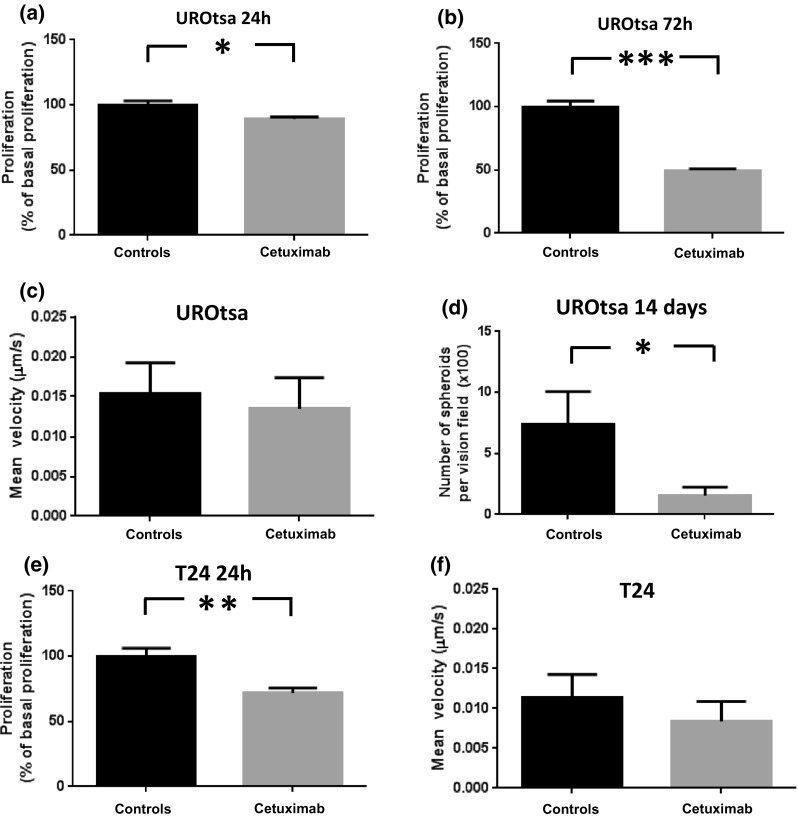
Urothelial proliferation (% of basal responses) in 24-h (**a**) and 72-h (**b**) UROtsa cultures, migration velocity in UROtsa cultures (**c**), number of round cells in 14-day UROtsa cultures (**d**), urothelial proliferation in 24-h T24 cell cultures and migration velocity in T24 cultures in the absence and presence of cetuximab (1.5 μM). *p < 0.05, **p < 0.01 and ***p < 0.001. Vertical bars represent the SEM

T24 and UROtsa cells migrated freely and formed frequent cell to cell interactions in the migration analysis. T24 cells divided more frequently than UROtsa cells (Fig. 2a, b). Even though mitosis occurred less frequently in UROtsa cells than in T24 cells, UROtsa cells stayed in the rounded shape longer than T24 cells (Fig. 2c, d). T24 normally underwent mitosis following forming the rounded shape, while UROtsa cells rarely underwent mitosis after this event. Incubation with cetuximab (1.5 μM) inhibited formation of the rounded shape in UROtsa and inhibited the number of attached T24 cells at time point 120 min (*p* < 0.05; *n* = 3–4; Fig. 2a, b). While UROtsa cells divided with regular mitosis, T24 cells also divided with tripolar mitoses, i.e., one cell dividing into three daughter cells (Fig. 2e). Incubation with cetuximab inhibited proliferation in both the UROtsa and the T24 cell line in 24-h cultures (*p* < 0.01–0.05; *n* = 4; Fig. 3a, e). In 72-h cultures, the inhibiting effect of cetuximab on proliferation of UROtsa was even more pronounced (*p* < 0.001; *n* = 8; Fig. 3b). Cetuximab inhibited also the number of three-dimensional cysts in UROtsa grown three-dimensionally for 14 days (*p* < 0.05; *n* = 5; Fig. 3d). While proliferation was affected by cetuximab, the urothelial migration velocity was instead not affected by EGFr blockade (n.s.; *n* = 3–4; Fig. 3c, f).

### Western blot analyses

**Fig. 4:**
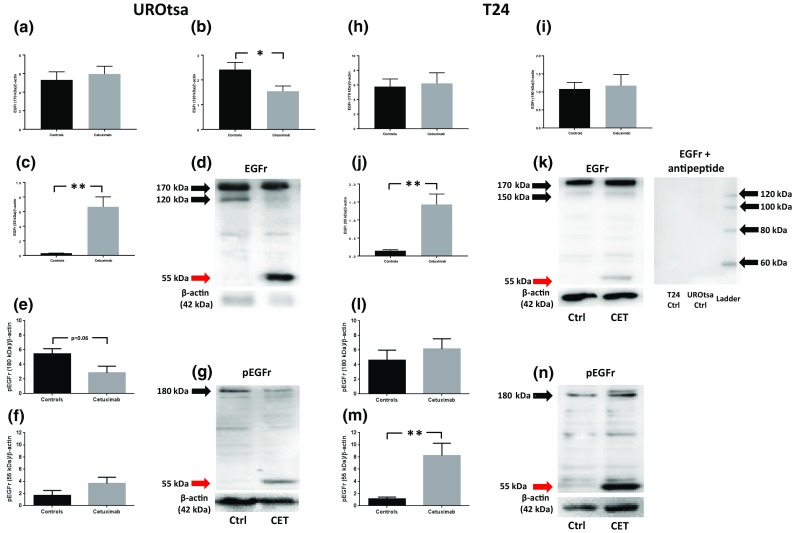
Western blot analyses of EGFr expressions in UROtsa (**a**–**c**) and in T24 (**h**–**j**); representative graphs in **d**, **k**, respectively, and pEGFr in UROtsa (**e**, **f**) and in T24 (**l**–**m**); representative graphs in **g**, **n**, respectively. Black arrows indicate EGFr and pEGFr, respectively, while red arrows indicate fragments of protein at 55 kDa. Membrane incubated with anti-EGFr in the presence of EGFr peptide is displayed in 4 k. *p < 0.05 and **p < 0.01. Vertical bars represent the SEM

**Fig. 5:**
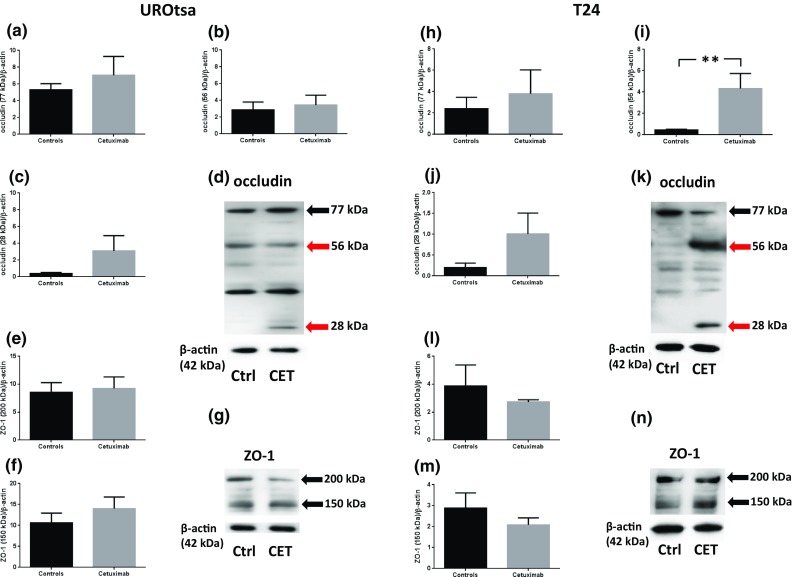
Western blot analyses of occludin expressions (77, 56 and 28 kDa in UROtsa (**a**–**c**) and in T24 (**h**–**j**); representative graphs in **d**, **k**, respectively) and ZO-1 (200 and 150 kDa in UROtsa (**e**–**f**) and in T24 (**l**–**m**); representative graphs in **g**, **n**, respectively). Black arrows indicate occludin and ZO-1, respectively, while red arrows indicate fragments of occludin. **p < 0.01. Vertical bars represent the SEM

EGFr was expressed in UROtsa (170 and 120 kDa) and in T24 (170 and 150 kDa). The phosphorylated form of EGFr (pEGFR; 180 kDa) was expressed in both UROtsa and T24 cells (Fig. 4). Treating UROtsa with cetuximab (1.5 μM) for 3 days resulted in a breakdown of EGFr, where the band of 120 kDa was less expressed (*p* < 0.05; *n* = 6) and a band at 55 kDa appeared (*p* < 0.01; *n* = 6; Fig. 4a–d) and strong tendencies to a down-regulation of pEGFr also occurred (*p* = 0.06; *n* = 5–6; Fig. 4e–g). In T24, incubation with cetuximab resulted in the appearance of a EGFr 55-kDa band (*p* < 0.01; *n* = 6; Fig. 4j–k) and a pEGFr 55-kDa band (*p* < 0.01; *n* = 6; Fig. 4m–n). Incubating membranes with anti-EGFr in the presence of EGFr peptide resulted in the disappearance of EGFr bands in UROtsa (170 and 120 kDa) and in T24 (170 and 150 kDa; Fig. 4k). In view of that cetuximab inhibited the formation of cysts in UROtsa, we wanted to explore whether cetuximab affected the expression of gap junction proteins. Western blot analyses showed the expression of occludin (77 kDa; 56 kDa; Fig. 5a–d in UROtsa; Fig. 5h–k in T24) and zonula occludens 1 (ZO-1; 200 kDa; 150 kDa; Fig. 5e–g in UROtsa; Fig. 5l–n in T24). Challenging urothelial cells with cetuximab (1.5 μM) resulted in a breakdown in occludin in T24 (bands at 56 and 28 kDa; *p* < 0.01; *n* = 6; Fig. 5i–k), while in UROtsa a similar band at 28 kDa as in T24 also tended to appear in response to cetuximab challenge (n.s.; *p* = 5–6; Fig. 5c, d). The expression of ZO-1 was unaffected by cetuximab in both UROtsa and T24 (n.s.; *p* = 5–6; Fig. 5e–g and 5l–m, respectively).

## Discussion

The knowledge of epithelial function has in many reports relied on studies on cells grown with conventional cell cultures, i.e., in two-dimensional cell cultures. A three-dimensional cell culture may, however, be closer to the in vivo situation where cells may grow in any direction. In the present study, we assessed how EGFr affects growth of urothelial cells by using a three-dimensional cell culture technique allowing cells to grow in all directions. We assessed EGFr functions in two urothelial cell lines where the UROtsa cell line is derived from normal epithelium and the T24 cell line is derived from a malignant bladder tumor [12, 13]. When grown three-dimensionally, the UROtsa cell line formed multi-cellular spheroids, while the T24 cell line did not. The UROtsa cells forming the spheroids did, however, not seem to polarize creating a hollow space inside the spheroid as demonstrated, for example, in kidney epithelial cells grown three-dimensionally [15]. We identified that the UROtsa cells expressed CK20, while laminin expression was very weak and CK17 was not expressed. Superficial urothelial cells express CK20, while intermediate and basal cells express CK17 as demonstrated in the rat urothelium [16]. We showed the presence of occludin in UROtsa, which is in line with previous reports showing the expression of gap junction in UROtsa [17]. Previous studies have shown that occludin is expressed by tight junctions in the umbrella cell layer [18]. The scope of our study was not to fully characterize the phenotype of the UROtsa and T24 cell lines, and therefore, immunohistochemistry of only a small number of markers for umbrella cells and basal/intermediate cells was performed. Based on our immunohistochemical results and from the literature, the UROtsa cell line seems to resemble cells not derived from the basal cell layer [17]. The UROtsa cell line has been used in models for urothelial transformation [19, 20]. Since the UROtsa cell line therefore appears differentiated, it may be difficult for further differentiation induced by external inputs. The UROtsa spheroids may therefore be constituted only by a homogenous urothelial cell phenotype. Under the same growing conditions as UROtsa cells, T24 cells grew dynamically but grew instead individually and did not form any spheroids. Since the two cell lines with contra-polarized phenotypes differed in the growing pattern, our cell culture technique may therefore be valuable to study urothelial function and transformation.

In line with previous studies, we show the expression of EGFr in UROtsa [21]. We found evidence that EGFr affects proliferation, the three-dimensional formation of the urothelium and the regulation of tight junction proteins. Blockade of EGFr also induced cleavage of EGFr and pEGFr. Our findings are in line with findings showing that EGFr inhibitors reduce phosphorylation of EGFr in lung cancer cell lines [22]. EGFr also modulates the formation of cellular cysts in polycystic kidney disease, which further supports the results in our study [23].

Mitotic cell rounding is an important cellular event for the mitosis process but also for regulating lumen formation [14, 24, 25]. Mitotic cell rounding also facilitates epithelial invagination, an event important for morphogenesis [26]. While the malignant T24 cell line always underwent mitosis after mitotic rounding, the normal UROtsa cell line often stayed in a rounded shape without undergoing mitosis. This might indicate that mitotic rounding may contribute also to the development of the three-dimensional structure of normal urothelium. To note, cetuximab inhibited partly the number of cells undergoing mitotic rounding and inhibited potently the formation of UROtsa cysts. This further strengthens the hypothesis that EGFr contributes to the three-dimensional structure of the urothelium. Cell rounding may also be a sign of apoptosis [27]; however, during the studied time window (2–15 h after plating), we did not observe rounded cells undergoing apoptosis or other apoptotic cellular changes.

EGFr may generate the redistribution and expression of tight junction proteins in studies on canine renal epithelial cell lines and cell lines derived from lung and pancreatic cancers [28–30]. Since our findings showed that UROtsa cyst formations were decreased by blocking EGFr, we wanted to explore whether tight junction proteins were affected by EGFr blockade. EGFr blockade induced a prominent cleavage of occludin in T24, while ZO-1 was not affected. In cetuximab-treated T24 cells, a band of 56 kDa appeared. In UROtsa, the 56-kDa band was already present in control cells and the expression was not changed upon exposure of cetuximab. Although the patterns in expression of the 56-kDa bands were different in the two urothelial cell lines, a band of 28 kDa appeared both in the T24 and the UROtsa cell line. Previous studies show that in apoptotic epithelial cells, occludin is cleaved into two fragments, i.e., a fragment of 55 kDa and a fragment of 31 kDa [31]. Therefore, our findings of the changed expression of occludin may be the result of apoptosis and not necessarily the result of changes in tight junction dynamics due to the influence of EGFr inhibition. Similar to our findings, ZO-1 was not affected under apoptosis in the study by Bojarski et al. [31]. Studies show that EGF activation in MDCK cells may regulate the expression of different claudins, proteins that also build up tight junctions [32]. The mechanisms behind the lower number of UROtsa cysts upon EGFr blockade remain elusive, however, by distorting the formation of tight junctions may as a consequence jeopardize the formation of cellular cysts.

In conclusion, our study shows that the three-dimensional way of culture may facilitate the study of urothelial differentiation and transformation. EGFr seems to be important for both urothelial proliferation and the formation of the three-dimensional structure of the epithelium.
